# Brain activation during standing balance control in dual-task paradigm and its correlation among older adults with mild cognitive impairment: a fNIRS study

**DOI:** 10.1186/s12877-024-04772-1

**Published:** 2024-02-10

**Authors:** Guocai Xu, Mian Zhou, Yan Chen, Qipeng Song, Wei Sun, Jiangna Wang

**Affiliations:** 1 College of Sports and Health, Shandong Sport University, Jinan, Shandong China; 2 Rehabilitation Medicine Department, Weishan People’s Hospital, Jining, Shandong China

**Keywords:** MCI, Prefrontal cortex, Postural control, Balance, Functional near-infrared spectroscopy

## Abstract

**Background:**

This study aimed to compare the balance ability and functional brain oxygenation in the prefrontal cortex (PFC) among older adults with mild cognitive impairment (MCI) under single and dual tasks, and also investigate their relationship. Neural regulatory mechanisms of the brain in the MCI were shed light on in balance control conditions.

**Methods:**

21 older adults with MCI (female = 12, age: 71.19 ± 3.36 years) were recruited as the experimental group and 19 healthy older adults (female = 9, age: 70.16 ± 4.54 years) as the control group. Participants completed balance control of single task and dual task respectively. Functional near-infrared spectroscopy (fNIRS) and force measuring platform are used to collect hemodynamic signals of the PFC and center of pressure (COP) data during the balance task, respectively.

**Results:**

The significant Group*Task interaction effect was found in maximal displacement of the COP in the medial-lateral (ML) direction (D-ml), 95% confidence ellipse area (95%AREA), root mean square (RMS), the RMS in the ML direction (RMS-ml), the RMS in the anterior-posterior (AP) direction (RMS-ap), sway path (SP), the sway path in the ML direction (SP-ml), and the sway path in the AP direction (SP-ap). The significant group effect was detected for five regions of interest (ROI), namely the left Brodmann area (BA) 45 (L45), the right BA45 (R45), the right BA10 (R10), the left BA46 (L46), and the right BA11 (R11). Under single task, maximal displacement of the COP in the AP direction (D-ap), RMS, and RMS-ap were significantly negatively correlated with R45, L45, and R11 respectively. Under dual task, both RMS and 95%AREA were correlated positively with L45, and both L10 and R10 were positively correlated with RMS-ap.

**Conclusion:**

The MCI demonstrated worse balance control ability as compared to healthy older adults. The greater activation of PFC under dual tasks in MCI may be considered a compensatory strategy for maintaining the standing balance. The brain activation was negatively correlated with balance ability under single task, and positively under dual task.

**Trial registration:**

ChiCTR2100044221, 12/03/2021.

## Background

MCI is a transitional stage between normal aging and Alzheimer’s disease [1]. So far, 16% of people over the age of 70 have been diagnosed with MCI [2]. And develop Alzheimer’s disease at 4–15% a year [3]. It is reported that MCI has been identified as an important risk factor for instability and falls in older adults [4], and the incidence of falls in older adults with MCI is twice as high as that in healthy older adults compared with the older adults with complete cognition [5].

Both balance control and cognitive function play important roles in preventing falls and injuries in older adults [6]. MCI is characterized by objective memory impairment over age [1]. Compared with healthy older adults, the cognitive function of older adults with MCI decreased, including attention, language fluency, and name recall [7]. At the same time, older adults with MCI showed defects in balance ability and posture control ability [8], Compared with healthy older adults, there was an increase in postural sway in standing balance control in older adults with MCI, especially a significant decrease in balance in medial-lateral direction [9]. Balance disorders are positively associated with cognitive impairment, and both increase the risk of falls [10]. While it is common to perform cognitive tasks while standing or walking in daily life, the ability of older adults with MCI to allocate limited attention resources to posture control decreases, which will lead to a further increase in the risk of falls [11].

Cognitive-motor control dual-task paradigm is often used to study the interaction between the allocation of brain cognitive resources and neuromuscular behavior control [12]. According to capacity-sharing theory, cognitive resources are limited in capacity. If the two tasks performed together exceed the available cognitive capacity, then the capacity to perform both tasks optimally will be insufficient, and the performance of either or both tasks will deteriorate [13]. For example, performing cognitive tasks while standing reduces postural stability, and older adults are more likely to wobble when performing cognitive-postural control dual tasks than performing postural control alone [14]. Compared with healthy older adults, the older adults with MCI showed higher dual-task costs during level walking, and poor dual-task performance was associated with the transformation of older adults with MCI into dementia, which was an important predictor of future falls [15]. However, few studies investigate the balance ability under dual tasks in the MCI.

The brain area responsible for executive function is mainly located in the PFC, which is closely related to the dorsolateral PFC [16]. The relationship between executive function and movement has been extensively studied using the dual-task paradigm [12]. Cognitive decline associated with executive function may be a contributing factor to postural instability and falls [17]. A fNIRS study, revealing the compensatory mechanism of PFC activity for standing balance in older adults, found that older adults increased PFC activation compared to young adults during dual-task balance. PFC activation compensates for sensorimotor deficits to maintain stability [18]. However, at present, there is a lack of research on the relationship between static standing balance and cognitive function in MCI, and there are relatively few reports on the neuroregulatory mechanism of the dual-task paradigm affecting static postural control in MCI.

The present study aims to explore the characteristics and correlation of standing balance control ability and prefrontal oxygenation level in older adults with MCI under single-task and dual-task paradigms compared with healthy older adults. This study might help to understand the neurobiological mechanism of cognition and balance performance and also provide a theoretical basis for the formulation of targeted prevention and clinical rehabilitation intervention strategies. The following hypotheses are formulated: (1) While performing dual tasks, the standing balance control ability of the older adults with MCI was worse than that of the healthy older adults, and also the older adults with MCI required more PFC activation. And (2) the balance ability of healthy older adults and older adults with MCI is positively correlated with the activation level of PFC.

## Methods

### Participants

In this study, 21 older adults over 65 years old diagnosed with MCI and 19 healthy older adults were recruited from the local community as a control group. The identification of MCI was based on the consensus criteria [19] that included the presence of subjective memory complaints from the patient and family, objective memory impairment (assessed using the Mini-Mental State Examination [MMSE] and Montreal Cognitive Assessment [MoCA]), preserved general intellectual function (assessed clinically), absence of significant functional impairment, and absence of clinical dementia [19]. Additionally, participants needed to score 0.5 on the Clinical Dementia Rating Scale (CDR) [11]. The inclusion and exclusion criteria of participants were as follows [11, 20]: inclusion criteria for the MCI group were (a) clinically diagnosed with MCI; (b) aged 65 years and above; (c) and able to walk independently without a gait aid; (d) MoCA scale score < 26. Inclusion criteria for the control group were 65 years and older, no cognitive impairment, no functional impairment, ability to walk independently without gait assistance, and a score of ≥26 on the MoCA scale. Exclusion criteria for both groups were (a) Parkinson’s disease or any neurological disease with motor deficits (e.g., stroke, epilepsy); (b) musculoskeletal disorders or a history of knee or hip replacement surgery that affected gait performance; (c) use of psychotropic medications that affected motor performance (eg, neuroleptics, benzodiazepines), or active major depression; (d) severe uncorrected visual or auditory impairment.

### Sample size calculation

The prior power analysis (G*power version 3.1) indicated that a minimum of 22 participants were needed to obtain the alpha level of 0.05 and the power level of 0.80 based a on previous study [21], which reported that a significant main effect of condition on HbO_2_ values, η^2^_p_ = 0.289. To detect statistically significant differences, 40 participants were recruited.

This study was approved by the Ethics Committee of Shandong Sport University (approval number: 2021006) and complied with the Declaration of Helsinki. All participants signed the informed consent form before the experiment. There were no significant differences in age, height, weight, or years of education between the MCI and control group (Table 1).

**Table 1:** Participants’ basic information (mean ± standard deviation)

	Control group	MCI group	*p* value
**Age (year)**	70.16 ± 4.54	71.19 ± 3.36	0.415
**Sex (male/female)**	10/9	9/12	0.536
**Height (cm)**	163.53 ± 6.63	159.71 ± 7.29	0.055
**Weight (kg)**	64.46 ± 8.07	63.61 ± 10.04	0.771
**Years of education (year)**	6.74 ± 3.40	6.05 ± 3.31	0.52
**MoCA**	26.47 ± 0.90	18.33 ± 2.97	< 0.001

### Measures and procedures

#### Balance protocol

Standing balance control was performed in the Sports Biomechanics Laboratory. Each participant performs two tasks: (1) maintain a natural standing balance condition (single task), and (2) standing balance condition with the addition of a cognitive task (dual task).

In each task, the participants were required to stand barefoot with feet placed in parallel, with a heel midpoint distance of 20 cm [20], arms drooping naturally on both sides, while focusing on a black point placed at eye-level approximately 1 meter in front of the participants. Each standing balance task was required to remain still for at least 60 s [21], repeat the effective test 3 times, and rest for 1 minute after each test. The cognitive task requires participants to perform a sequence of subtracting 3 from a random number between 400 and 500. The force measuring platform (Kistler, 9281CA, 60 cm × 90 cm × 10 cm) was used to collect the COP displacement data of the balance task, and the acquisition frequency was 1000 Hz. Each task was demonstrated to the participants to ensure familiarity with the experimental procedure before the experiment began. The data collection was conducted synchronously during the standing balance and fNIRS tests.

#### Functional near-infrared spectroscopy test

**Fig. 1:**
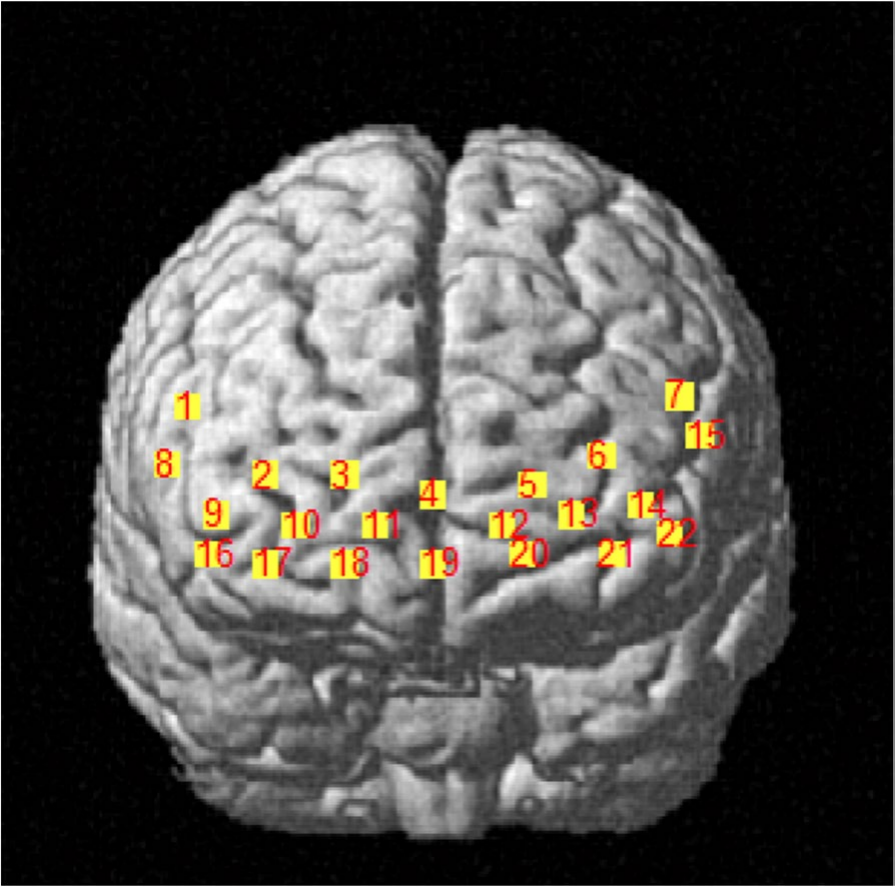
Distribution map of 22 channels

A portable near-infrared imaging system (LIGHTNIRS, Shimadzu Corp., Kyoto, Japan) was used to measure the PFC hemodynamic response via laser diodes with three wavelengths of 780 nm, 805 nm, and 830 nm (sampling frequency 13.3 Hz). The device consisted of 16 optodes with 8 light emitters and 8 light detectors (total 22 channels, as shown in Fig. 1). The distance between the emitter and detector was 30 mm. During the experiment, participants carried fNIRS while performing balance tasks and wore a whole-head fiber holder with the standard head landmarks determined according to the international 10/10 system. A 3D digitizer (FASTRAK, Polhemus, Vermont, USA) was used to determine MNI (Montreal Neurological Institute) coordinates [22]. The PFC was divided into 8 ROIs based on Brodmann areas [23].

Channels 2, 3, 4, 11, and 19 corresponded to the right frontopolar cortex (R-FPC) and belonged to the right BA10. Channels 5, 6, 12, 13, and 21 corresponded to the left frontopolar cortex (L-FPC) and belonged to the right BA10. Channels 9 and 16 corresponded to the right dorsolateral prefrontal cortex (R-DLPFC) and belonged to the right BA46. Channels 14 and 22 corresponded to the left dorsolateral prefrontal cortex (L-DLPFC) and belonged to the left BA46. Channels 1 and 8 corresponded to the right ventrolateral prefrontal cortex (R-VLPFC) and belonged to the right BA45. Channels 7 and 15 corresponded to the left ventrolateral prefrontal cortex (L-VLPFC) and belonged to the left BA45. Channels 10, 17, and 18 corresponded to the right orbitofrontal cortex (R-OFC) and belonged to the right BA11. Channel 20 corresponded to the left orbitofrontal cortex (L-OFC) and belonged to the left BA11.

Using the modified Beer-Lambert law, optical density was converted to blood oxygen concentration, including oxyhemoglobin concentration (HbO_2_), deoxyhemoglobin concentration (HHb), and total hemoglobin concentration (HbT). HbO_2_ was selected to characterize hemodynamic changes in the PFC during the balance task conditions because HbO_2_ is the most sensitive to locomotion-related changes in regional cerebral blood flow and individual differences in Hb are considerable in task-related changes in older adults. Furthermore, there are larger change amplitude and better signal-to-noise ratio in HbO_2_ [10]. In addition, using one index applicable for task-related hemodynamic changes reduced the comparison number and refrained from increasing the probability of Type I error.

### Data processing

#### Balance parameters

The COP data were low-pass filtered (Butterworth) with a cut-off frequency of 6 Hz [24]. The parameters were calculated as follows:

The maximal displacements of COP in the anterior-posterior (AP; D-ap) and medial-lateral (ML; D-ml) directions.

Root mean squared (RMS) distance: The RMS distance of the COP time series (RMS-ap and RMS-ml) to the average COP position is calculated as the average distance to the average COP position. The algorithm used to calculate the root mean square distance in RMS-ap is shown in formula (1):

1 $${\textrm{RMS}}_{\textrm{AP}}=\frac{1}{\textrm{N}}\sum {\left[\textrm{AP}{\left(\textrm{n}\right)}^2\right]}^{1/2}$$

95% confidence ellipse area (95%AREA): refers to the largest rectangular area surrounded by the swing trajectory of the center of gravity, reflecting the swing range of the body’s center of gravity. It is the 95% confidence interval that contains approximately 95% of the COP data points. The larger the corresponding envelope area, the larger the swing range and the worse the balance ability of the human body. The envelope area can comprehensively reflect the degree of balance obstacles, and the calculation formula is shown in (2):

2 $$\textrm{S}=\left(\textrm{Xmax}-\textrm{Xmin}\right)\times \left(\textrm{Ymax}-\textrm{Ymin}\right)\times \uppi$$

Xmax and Ymax are the maximum values of COP on the X and Y axes, and Xmin and Ymin are the lowest values of COP on the X and Y axes.

Sway Path (SP): refers to the length of the path taken by the COP data points, just as the COP data points can be stretched into a line and the length of the line can be measured. Reflects the speed at which the center of gravity swings. The larger the indicator, the more obvious the shaking, and the worse the ability of the human body to control the shaking of the center of gravity.

SP-ml refers to the total length of the COP path in the ML direction, and SP-ap refers to the total length of the COP path in the AP direction [25].

#### fNIRS parameters

HbO_2_ data were processed using MATLAB-based NIRS_SPM time series analysis (R2013b, The MathWorks, Inc., Natick, Massachusetts, USA). High-frequency noise was reduced/ removed by a low-pass filter based on the canonical hemodynamic response function (cut-off frequency 0.15 Hz) [10]. With the wavelet minimum description length algorithm [26], the fNIRS measurement signals were detrended to remove artifacts caused by breathing, heartbeat, vasomotor, and other movement-related disturbances. The average HbO_2_ concentration within 5 s before the balance test began was calculated for the baseline correction on the signal to obtain the HbO_2_ concentration change values (△HbO_2_).

After data preprocessing, the △HbO_2_ of the three tests for each task was superimposed and averaged to obtain the time-series changes of all channels of the individual under the two tasks. The data were averaged across all channels in the ROIs and consequently across all participants. The MATLAB-based NIRS_SPM toolkit was used to perform a general linear model (GLM) analysis to obtain the activation of brain areas. Specifically, GLM decomposes the observed signal as follows [27]:

3 $$\textrm{y}\left(\textrm{t}\right)=\sum_{\textrm{i}=1}^{\textrm{n}}\textrm{xi}\left(\textrm{t}\right)\times \upbeta \textrm{i}+\upvarepsilon \left(\textrm{t}\right)$$

βi is represented as the degree to which the fNIRS signal can be predicted or interpreted by the experimental condition i. The greater the value of βi, the higher the brain activation level under condition i.

#### Statistical analyses

SPSS20.0 (IBMS, NY, USA) is used for statistical analysis of the data. Three successful trials data were averaged for data analysis. The normality of all the outcomes was tested with the Shapiro-Wilk test. We compared difference in balance and PFC activation parameters with two-way multivariate analysis of variance (MANOVA), using groups (control group and MCI group), tasks (single-task and dual-task) as fixed factors, interactions between group and tasks. If a significant interaction was detected, the Bonferroni adjusted t-test method was conducted for post hoc comparisons. Partial eta squared (η^2^_p_) was used to represent the effect size of main effect and interaction of MANOVA. The thresholds for Partial eta squared were as follows: 0.01–0.06, small; 0.06–0.14, moderate; > 0.14, large [28]. Pearson correlation analysis was used to determine the relationship between brain activation parameters and balance ability parameters. The thresholds for the correlation coefficient (r) were as follows: 0–0.1, trivial; > 0.1–0.3, weak; > 0.3–0.5, moderate; > 0.5, strong [29]. The significance level was set to *P* < 0.05.

## Result

### Balance parameters

**Fig. 2:**
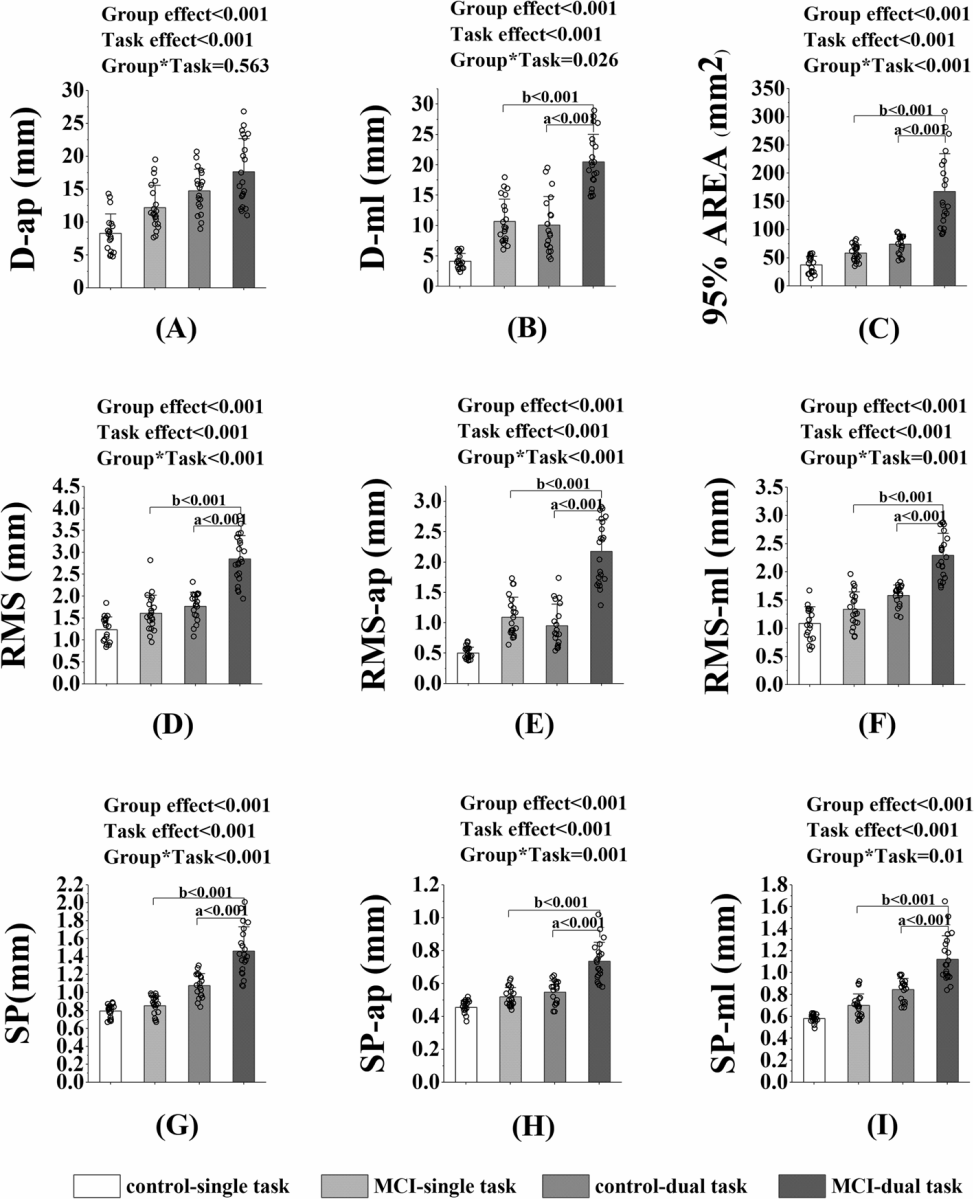
The balance ability under single task and dual tasks in the control and MCI groups. Note. D-ap = the maximal displacement of COP in the AP direction; D-ml = the maximal displacement of COP in the ML direction; RMS = Root mean squared; RMS-ml = Root mean squared in the ML direction; RMS-ap = Root mean squared in the AP direction; 95%AREA = 95% confidence ellipse area; SP = Sway Path; SP-ml = Sway Path in the ML direction; SP-ap = Sway Path in the AP direction. a, significant difference between groups. b, significant difference between tasks. *p < 0.05

Two-way MANOVA showed that the significant Group*Task interaction effect was found in D-ml (*p* = 0.026, η^2^_p_ = 0.064), 95%AREA (*p* < 0.001, η^2^_p_ = 0.200), RMS (*p* < 0.001, η^2^_p_ = 0.166), RMS-ml (*p* = 0.001, η^2^_p_ = 0.130), RMS-ap (*p* < 0.001, η^2^_p_ = 0.164), SP (*p* < 0.001, η^2^_p_ = 0.201), SP-ml (*p* = 0.01, η^2^_p_ = 0.085),and SP-ap (*p* = 0.001, η^2^_p_ = 0.145). Post-hoc comparisons showed that in the MCI group, all the above parameters were significantly higher under dual task compared to those under the single task (all *p* < 0.001); while no significant difference was found between single and dual task in the control group. Under dual tasks, all the above parameters were significantly higher in the MCI group compared to those in the control group (all *p* < 0.001). No significant Group*Task interaction effect was found (*p* = 0.563, η^2^_p_ = 0.004), while significant group effect (*p* < 0.001, η^2^_p_ = 0.172) and task effect (*p* < 0.001, η^2^_p_ = 0.390) were detected for D-ap. Similarly, significant group effect was found in D-ml (*p* < 0.001, η^2^_p_ = 0.566), RMS (*p* < 0.001, η^2^_p_ = 0.458), RMS-ml (*p* < 0.001, η^2^_p_ = 0.389), RMS-ap (*p* < 0.001, η^2^_p_ = 0.622), 95%AREA (*p* < 0.001, η^2^_p_ = 0.388), SP (*p* < 0.001, η^2^_p_ = 0.320), SP-ml (*p* < 0.001, η^2^_p_ = 0.368), and SP-ap (*p* < 0.001, η^2^_p_ = 0.410). Significant task effect was also found in D-ml (*p* < 0.001, η^2^_p_ = 0.529), RMS (*p* < 0.001, η^2^_p_ = 0.555), RMS-ml (*p* < 0.001, η^2^_p_ = 0.593), RMS-ap (*p* < 0.001, η^2^_p_ = 0.540), 95%AREA (*p* < 0.001, η^2^_p_ = 0.508), SP (*p* < 0.001, η^2^_p_ = 0.654), SP-ml (*p* < 0.001, η^2^_p_ = 0.636), and SP-ap (*p* < 0.001, η^2^_p_ = 0.507) (Fig. 2).

### PFC activation parameters

Two-way MANOVA showed that no significant Group*Task interaction effect was found in the eight ROI regions, while significant group effect was detected for five ROI regions, namely L45 (*p* = 0.019, η^2^_p_ = 0.071), R45 (*p* = 0.003, η^2^_p_ = 0.109), R10 (*p* = 0.029, η^2^_p_ = 0.060), L46 (*p* = 0.003, η^2^_p_ = 0.109), and R11 (*p* = 0.010, η^2^_p_ = 0.084). No significant task effect was found in the eight ROI regions (Table 2).

**Table 2:** Activation levels of each ROI region in the task condition in the control and MCI groups

ROI (μmol/L)	Control group		MCI group		Group		Task		Group*Task	
	Single task	Dual task	Single task	Dual task	p	η^2^_p_	p	η^2^_p_	p	η^2^_p_
L45	−2.64 ± 5.10	0.72 ± 6.92	1.71 ± 5.97	2.65 ± 5.20	0.019	0.071	0.103	0.035	0.356	0.011
R45	−0.85 ± 6.45	0.19 ± 6.93	4.79 ± 6.76	3.76 ± 6.84	0.003	0.109	1.000	0.000	0.496	0.006
L10	0.45 ± 5.91	1.31 ± 5.79	3.11 ± 7.00	2.62 ± 6.11	0.085	0.042	0.651	0.004	0.421	0.006
R10	−1.28 ± 4.88	0.89 ± 5.96	2.32 ± 5.52	2.67 ± 5.18	0.029	0.060	0.301	0.014	0.453	0.008
L46	−1.91 ± 7.14	0.65 ± 7.50	4.45 ± 6.43	2.70 ± 7.30	0.003	0.109	0.878	0.000	0.347	0.012
R46	0.27 ± 9.06	−0.62 ± 7.86	4.12 ± 8.70	2.16 ± 5.58	0.065	0.044	0.631	0.008	0.762	0.001
L11	−2.48 ± 8.67	−0.42 ± 12.37	3.59 ± 15.22	2.87 ± 6.37	0.066	0.044	0.790	0.001	0.582	0.004
R11	−2.55 ± 8.35	1.11 ± 6.99	3.12 ± 10.27	5.84 ± 8.99	0.010	0.084	0.914	0.033	0.810	0.001

L45 = the left ventrolateral prefrontal cortex (L-VLPFC) and belonged to the left BA45. R45 = the right ventrolateral prefrontal cortex (R-VLPFC) and belonged to the right BA45. L10 = the left frontopolar cortex (L-FPC) and belonged to the right BA10. R10 = the right frontopolar cortex (R-FPC) and belonged to the right BA10. L46 = the left dorsolateral prefrontal cortex (L-DLPFC) and belonged to the left BA46. R46 = the right dorsolateral prefrontal cortex (R-DLPFC) and belonged to the right BA46. L11 = the left orbitofrontal cortex (L-OFC) and belonged to the left BA11. R11 = the right orbitofrontal cortex (R-OFC) and belonged to the right BA11. Statistically significant differences between groups (*p* < 0.05)

### Correlation between PFC activation level and balance ability

**Fig. 3:**
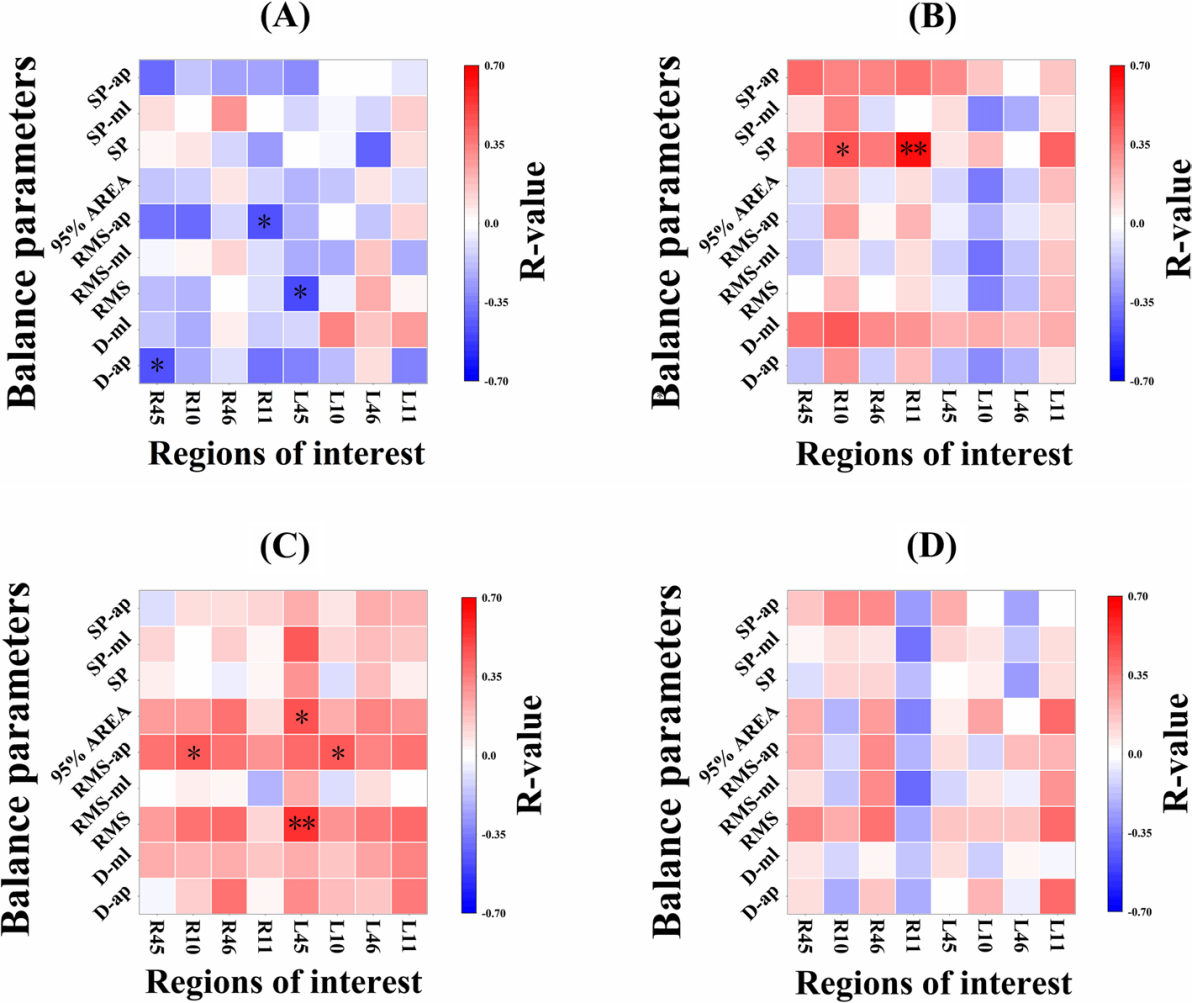
Correlation between PFC activation level and balance parameters under single task and dual task in the control and MCI groups. Note. **A** The correlation coefficient matrix of MCI group in single task. **B** The correlation coefficient matrix of control group in single task. **C** The correlation coefficient matrix of MCI group in dual task. **D** The correlation coefficient matrix of control group in dual task. The ordinate represents balance parameters, and the abscissa represents regions of interest. The color blocks represent the correlation coefficient between balance parameters and regions of interest. The range of − 0.7–0 for the color card represents a negative correlation, and the range of 0–0.7 represents a positive correlation. The red color represents greater correlation coefficient and strong positive correlation, and the blue color represents greater correlation coefficient and strong negative correlation. D-ap = the maximal displacement of COP in the AP direction; D-ml = the maximal displacement of COP in the ML direction; RMS = Root mean squared; RMS-ml = Root mean squared in the ML direction; RMS-ap = Root mean squared in the AP direction; 95%AREA = 95% confidence ellipse area; SP = Sway Path; SP-ml = Sway Path in the ML direction; SP-ap = Sway Path in the AP direction. L45 = the left ventrolateral prefrontal cortex (L-VLPFC) and belonged to the left BA45. R45 = the right ventrolateral prefrontal cortex (R-VLPFC) and belonged to the right BA45. L10 = the left frontopolar cortex (L-FPC) and belonged to the right BA10. R10 = the right frontopolar cortex (R-FPC) and belonged to the right BA10. L46 = the left dorsolateral prefrontal cortex (L-DLPFC) and belonged to the left BA46. R46 = the right dorsolateral prefrontal cortex (R-DLPFC) and belonged to the right BA46. L11 = the left orbitofrontal cortex (L-OFC) and belonged to the left BA11. R11 = the right orbitofrontal cortex (R-OFC) and belonged to the right BA11. *p < 0.05, **p < 0.01

D-ap was significantly negatively correlated with R45 (moderate, r = − 0.468, *p* = 0.032); RMS was significantly negatively correlated with L45 (moderate, r = − 0.485, *p* = 0.026); RMS-ap was significantly negatively correlated with R11 (moderate, r = − 0.469, *p* = 0.032) during the single task in the MCI group. No significant correlation was detected between the rest of them (Fig. 3A). While the MCI group under dual task, both RMS (strong, r = 0.555, *p* = 0.009) and 95%AREA (moderate, r = 0.485, *p* = 0.037) were correlated positively with L45; both L10 (moderate, r = 0.454, *p* = 0.039) and R10 (moderate, r = 0.451, *p* = 0.040) were positively correlated with RMS-ap (Fig. 3C).

In the control group under single task, both R10 (moderate, r = 0.463, *p* = 0.046) and R11 (strong, r = 0.639, *p* = 0.003) were significantly positively correlated with SP (Fig. 3B). However, in the control group under dual task, none of the balance results were significantly correlated with PFC activation parameters (Fig. 3D).

## Discussion

This study compared the balance ability and functional brain oxygenation in the PFC among MCI and healthy older adults under single and dual tasks, and also investigated their relationship. Our results partially support the hypothesis that older adults with MCI have poorer balance control than healthy older adults.

The first hypothesis was demonstrated in the present study. Our results showed that under the dual tasks, the balance control ability of the older adults with MCI is significantly lower than that of the healthy older adults. And the PFC activation level of older adults with MCI was higher than that of healthy older adults, which concurred with previous studies investigating the decreased balance control ability in the MCI [30] and execution of dual-tasks increases PFC activation in older adults with MCI [31]. Balance control is a complex neuromuscular control process, that involves sensory detection of body movement, integration of sensorimotor information in the central nervous system (CNS), and appropriate programming and execution of neuromuscular responses [32]. Aging leads to the decline of physical and cognitive function in older adults, and more cognitive resources are needed to perform simple motor tasks in older adults [33]. However, compared to healthy older adults, the working memory and attention functions of older adults with MCI are impaired [7], and a decreased integration ability of sensorimotor information during multiple-task postural control [34]. Therefore, when performing dual tasks, older adults with MCI showed worse balance control than healthy older adults.

Interestingly, we observed that the PFC activation level of older adults with MCI was higher than that of healthy older adults in both single task and dual task **(**Table 2**)**. PFC is critical to a person’s ability to selectively allocate attention [35] and integrate visual and proprioceptive information [36] to maintain postural stability. Higher PFC activation is usually attributed to a decline in the efficiency of multi-sensory information processing in the aging brain, which leads to automatic impairment of movement, and leads to higher attention demand [37]. For older adults, PFC activation reflects an attempt to recruit a higher amount of limited neural resources to compensate for sensorimotor deficits to maintain balance control stability [18]. A previous study consistently found that increased PFC activity could compensate for damaged cortical circuits in other neuropsychiatric disorders to maintain a level of cognitive task performance comparable to that of healthy controls [38]. In this study, compared with healthy older adults, older adults with MCI have attention deficit [34] and postural control deficiency [8]. Structural and functional brain networks in MCI are both disrupted and associated with compromised cognitive performance [39], and these anatomical and functional changes can interfere with the balance control ability in MCI [32]. Therefore, older adults with MCI might need more PFC activation to maintain a standing balance.

It is worth noting that there was no task main effect on PFC activation in either two groups. We speculated that the attention resources of older adults with MCI are mainly used for balance maintenance under single task. While our balance control task was relatively simple [21] and not sufficient to pose a threat to postural control of older adults with MCI. Therefore, older adults with MCI can still handle the low-load tasks without overcompensation and thus do not exhibit over-activation of the PFC [40]. However, the balance control ability of older adults with MCI decreased in the dual task compared with the single task, the balance control ability of healthy older adults was not significantly disturbed by the dual task. We speculated that when disturbed by the dual-task, neither group of participants increased the activation of the PFC, but the balance control ability of the MCI can not be adequately maintained at the current level of PFC activation, while the healthy older adults can be maintained. This finding may be due to impaired working memory and attention function in older adults with MCI [7] and reduced ability to integrate sensorimotor information [34].

The second hypothesis was partially proven as follows: under single task, the balance parameters were correlated negatively with PFC activation parameters in older adults with MCI. That is, the higher the activation level of PFC, the better the balance ability (Fig. 3A). In contrast, the balance parameters were correlated positively with PFC activation parameters in healthy older adults (Fig. 3B). In previous studies, older adults with MCI need more PFC activation to participate in postural control than healthy older adults because of attention deficit [34] and postural control deficiency [8]. Therefore, for older adults with MCI, more PFC activation may be more conducive to the maintenance of balance [41]. As in this study, the better the balance, the higher the PFC activation level in older adults with MCI (Fig. 3A).

A fNIRS study consistently showed that when the level of PFC activation was lower, the performance of the healthy group was better, while that of the MCI group was on the contrary [42]. In contrast, when healthy older adults performed the single task, the better their balance ability, the lower their PFC activation level (Fig. 3B). We speculate that the differences in brain anatomy and function between the two groups might lead to the opposite results of the correlation analysis. Previous studies have shown that white matter content in older adults with MCI is appreciably different from those in healthy older adults [20]. And subcortical neuroanatomical changes in MCI, which are a central cholinergic deficit related to the loss of neurons in the nucleus basalis of Mynert and amyloid deposition and neurofibrillary tangles in the mesial temporal structure [32]. These changes in brain structure and function adversely affect the balance control system in older adults with MCI [32]. The activation of PFC is to compensate for the subcortical information processing deficiencies in older adults with MCI [18]. Therefore, older adults with MCI require more PFC activation to maintain a standing balance, that is, the higher the PFC activation, the better the balance.

However, our data shows that the PFC activation parameters of older adults with MCI were positively correlated with the balance parameters under the condition of the dual task, which was opposite to that of older adults with MCI under the condition of the single task (Fig. 3C). None of the balance parameters were significantly correlated with PFC activation parameters in healthy older adults (Fig. 3D). When performing dual tasks, the lower the level of PFC activation in older adults with MCI, the better the balance control ability; this is contrary to the correlation under single task (Fig. 3 A, C). The possible explanation is that there is an interference effect in the dual-task execution of older adults with MCI under the dual tasks, and the simultaneous tasks will interfere with each other, thus affecting the dual-task execution [43]. This is consistent with the central capacity sharing model [13]. When attention demand increases, PFC compensatory activities or neural compensation may increase and exceed available resources [13]. Therefore, when older adults with MCI performe dual tasks, multi-system aging (such as sensory or cognitive impairment, and reduced motor ability) means competition for common neural structures [44]. Under dual tasks, even if the PFC activation increases, the balance control ability decreases due to the interference of cognitive tasks.

Unlike the MCI, the healthy older adults did not observe the correlation between the PFC activation and balance control ability under the dual task (Fig. 3D). The single task seems to be a relatively simple balance control task for healthy older adults, which may not be challenging enough [21]. The task priority model [13] holds that when posture threat increases, older adults give priority to postural control rather than cognitive task performance. Therefore, even if the balance-cognitive dual task is performed at the same time, the interference of the cognitive task is not enough to disturb the balance control, attention resources are prioritized to deal with cognitive tasks. Another possible explanation is that older adults might transfer processing resources to other brain areas under dual tasks [45]. Since brain activity was measured only in a limited area of the PFC in this study, further study could investigate more brain areas for insight comprehension.

Our findings found that BA45, BA10, and R11 areas play an important role in the standing balance control task of older adults with MCI. BA45 area plays an important role in planning the high-level motion sequences, which not only participates in language processing but also participates in various tasks and content fields [36]. Previous studies have confirmed that the execution of various cognitive tasks and motor tasks could activate the BA45 area [46]. We also found that when performing single tasks, older adults with MCI need more activation of the BA45 area to maintain balance and stability. The BA10 area, the largest and most anterior area of the human PFC, is involved in multitasking, especially in selecting and maintaining higher internal objectives while performing other sub-objectives [47]. Previous studies have shown the functional separation between the medial and lateral surfaces of the BA10 area [48]. According to “multitasking” tests and functional neuroimaging experiments, the medial surface of the BA10 diverts attention to thoughts guided by sensory stimuli, while the lateral surface focuses on internally generated ideas [49]. Our study found that there was a significant positive correlation between bilateral BA10 activation and balance control in older adults with MCI when performing dual tasks. We speculated that both the medial and lateral activation of BA10 was evoked simultaneously due to the increase of cognitive task and balance difficulty at the same time, and we required participants to speak out the results of the calculation. The BA11 area is highly susceptible to age-associated changes, including disruption of frontostriatal circuits by diffuse white matter changes and gray matter atrophy [50]. Previous studies indicate that older adults exhibited significantly greater activation than young adults during motor imagery in R11 [51]. We also found that older adults with MCI need more activation of the R11 area to maintain balance control during single task.

The current research may be of great significance in strengthen the balance control strategy of older adults with MCI. The theory of brain plasticity holds that the brain can still show plasticity in the face of cognitive decline in old age [52]. Dual-task training can improve the speed of attention conversion, improve the ability of attention allocation between tasks, and make the allocation of cognitive resources among different tasks more coordinated [53]. Therefore, our study can be used to guide and formulate targeted task balance control therapy [53] and optimize alternative interventions such as non-invasive brain stimulation [52]. Up-regulation or down-regulation of the excitability of specific brain areas (such as BA45 and BA10) of PFC may have a certain effect on promoting balance control in older adults with MCI.

The current study has some limitations. First of all, we only assessed one static standing posture task and one cognitive task for the study, and did not quantify the difficulty level of the cognitive task and balance task. Secondly, this was a cross-sectional study, further studies could focus on the effect of dual-task training on balance and PFC activation in MCI. Finally, due to the limitation of experimental instruments, it is impossible to obtain more information about the cerebral cortex except for PFC.

## Conclusion

The MCI demonstrated worse balance control ability as compared to healthy older adults. The greater activation of PFC under dual tasks in MCI may be considered a compensatory strategy for maintaining the standing balance. The brain activation was negatively correlated with balance ability under single task, and positively under dual task. The current research results may have positive guiding significance for rehabilitation treatment, and special physical and non-physical interventions can be developed according to our results to improve the balance control of older adults with MCI.

## Data Availability

The datasets used and/or analysed during the current study available from the corresponding author on reasonable request.

## Abbreviations

MCI: Mild cognitive impairment
PFC: Prefrontal cortex
fNIRS: Functional near-infrared spectroscopy
COP: Center of pressure
MoCA: Montreal Cognitive Assessment
MMSE: Mini-Mental State Examination
ROI: Regions of interest
BA: Brodmann areas
RMS: Root mean squared
SP: Sway Path
AP: Anterior-posterior
ML: Medial-lateral
